# Automatic Method for Building Indoor Boundary Models from Dense Point Clouds Collected by Laser Scanners

**DOI:** 10.3390/s121216099

**Published:** 2012-11-22

**Authors:** Enrique Valero, Antonio Adán, Carlos Cerrada

**Affiliations:** 1 School of Computer Engineering, Universidad Nacional de Educación a Distancia (UNED), C/Juan del Rosal, 16, 28040 Madrid, Spain; E-Mail: ccerrada@issi.uned.es; 2 3D Visual Computing and Robotics Lab, Universidad de Castilla-La Mancha (UCLM), Paseo de la Universidad, 4, 13071 Ciudad Real, Spain; E-Mail: Antonio.Adan@uclm.es

**Keywords:** 3D modeling, B-rep models, laser scanners, 3D data processing

## Abstract

In this paper we present a method that automatically yields Boundary Representation Models (B-rep) for indoors after processing dense point clouds collected by laser scanners from key locations through an existing facility. Our objective is particularly focused on providing single models which contain the shape, location and relationship of primitive structural elements of inhabited scenarios such as walls, ceilings and floors. We propose a discretization of the space in order to accurately segment the 3D data and generate complete B-rep models of indoors in which faces, edges and vertices are coherently connected. The approach has been tested in real scenarios with data coming from laser scanners yielding promising results. We have deeply evaluated the results by analyzing how reliably these elements can be detected and how accurately they are modeled.

## 1. Introduction

During the last decade laser scanners have gained popularity in architecture, engineering, construction and facility management (AEC/FM). Other measurement methods such as total stations, measuring tapes and based-stereo-camera prototypes are too time-consuming or inaccurate compared to scanners, particularly in large-scale environments. Moreover the high density provided by a single scan (which could be over several million points) makes this technology very suitable for use in the 3D modeling of facilities.

Most of the time, the dense raw data provided from scanners are manipulated by a designer or processed by an engineer in order to create a simplified model of the scenario. This well known reverse engineering process is applied to 3D model creation. Thus, a simplified model provides a high-level representation of the scenario that ranges from a single CAD model, in which a wall is represented as a set of independent planar surfaces, to a Building Information Model (BIM), in which a wall is thought as a volumetric object composed by multiple surfaces with several relevant properties like color, material, cost, *etc.* Much of the emphasis in previous works has been on creating visually realistic non-parametric models rather than accurate parametric ones. Some examples in this category include methods by El-Hakim *et al.*[1], which is focused on indoor environments and Früh *et al.*[2] and Remondino *et al.*[3], which are focused on outdoor environments. In these cases, a great part of the modeling process is supervised by the modeler so that it cannot be said that models are obtained in an automatic manner.

In this paper we introduce a method that automatically yields Boundary Representation Models (B-rep) for indoors after processing dense point clouds collected by laser scanners from key locations through an existing facility. The 3D model then represents the current state of the building, what is named as the “as-is condition”. This model does not necessarily have to be coincident with either the designed (“as-designer condition”) or the built model (“as-built condition”). In fact, the facility could have lightly been modified or restored from its initial design. In other occasions, we cannot access to the design drawings or they merely do not exist. Thus, the automatic creation of “as-is models” of inhabited scenarios is a challenging research field which is gaining attention from different applications including architecture, engineering, robotics, *etc.*[4].

Although there are not many publications dealing with the automatic creation of 3D models, in the last years several interesting works related with some of the stages of this process can be found in the literature. A review for automatic reconstruction of as-built building information models can be found in [4]. The process of creating a single-semantic model can vary depending on the input and expected output. Generally, the automatic modeling process can be divided into three steps: data acquisition, data processing and data modeling, and most of the published papers concentrate on the second stage: data processing.

In this framework, 3D data processing means processing millions of unstructured 3D data in order to obtain higher level data structures. Several approaches that convert 3D data into high-level representations in buildings context can be found over the last years. We can here distinguish between proposals to detect and model single objects or particular parts of large scenarios and those than completely model indoors and outdoors.

As regards the first sort of proposals, one of the earlier works that obtain 3D models from laser scanner in a local space context is the one of Kwon *et al.*[5]. They introduce a set of algorithms for fitting sparse point clouds to a set of single volumetric primitives (cuboids, cylinders and spheres) which can be extended to groups of primitives belonging to the same object. An automated recognition/retrieval approach for 3D CAD objects in the construction context is presented in [6]. In [7] a semiautomatic method to match 3D existing models to data of industrial building steel structures is proposed. The author develops a variant of the ICP algorithm for data registration in order to recognize CAD models objects in large site laser scans. The method is semiautomatic because a coarse registration needs to be performed manually. The same author presents in [8] a plane based registration system for coarse registration of laser scanners data with 3D models in the context of AEC/FM industry. The approach is based on finding planes from the point cloud and matching them with the ones extracted from the 3D models. Nevertheless, the matching process is performed by hand. Other authors [9] propose the combination of point clouds, acquired by means of laser scanners, and photogrammetry in order to generate 3D models of a building under construction. The work of Rusu *et al.*[10] correctly recognizes and localizes relevant kitchen objects including cupboards, kitchen appliances, and tables. They interpret the improved point clouds in terms of rectangular planes and 3D geometric shapes. One of the innovations consists of including a novel multi-dimensional tuple representation for point clouds and robust, efficient and accurate techniques for computing these representations, which facilitate the creation of hierarchical object models.

Detailed models of part of the walls and building façades are obtained in [2,11–13]. In [11,12] the data processing goes from detecting windows through low data density regions to discover other data patterns in the façade. In [13] important façade elements such as walls and roofs are distinguished as features. Then, the knowledge about the features’ sizes, positions, orientations, and topology is introduced to recognize these features in a segmented laser point cloud. Früh *et al.*[2] develop a set of data processing algorithms for generating textured façade meshes of cities from a series of scans, corresponding to vertical 2D surfaces, obtained by a laser scanner. Thrun *et al.* developed a plane extraction method based on the expectation-maximization algorithm [14]. Other researchers have proposed plane sweep approaches to find planar regions [15,16]. Valero *et al.*[17] focus on the modeling of those linear moldings that typically surround doorways, windows, and divide ceilings from walls and walls from floors.

With respect to the automatic creation of complete indoor models, geometric surfaces or volumetric primitives can be fitted to a 3D point cloud to model walls, doors, ceilings, columns, beams, and other structures of interest. In its most simple format, the modeled primitives are annotated with labels (e.g., “wall”). In a higher modeling degree, spatial and functional relationships between nearby structures and spaces are established. Interesting developments can be seen in [18] and [19]. In these works, Adan *et al.* identify and model the main structural components of an indoor environment (walls, floors, ceilings, windows, and doorways) despite the presence of significant clutter and occlusion, which occur frequently in natural indoor environments, but they deal with rectangular rooms. Furthermore, in the present work we present an approach to identify walls in rooms with more complicated geometries. Okorn *et al.*[20] present an automated method for creating accurate 2D floor plan models of building interiors. They project the points onto a 2D ground plane and create a histogram of points density, from which line segments corresponding to walls are extracted using a Hough Transform. The first steps of our proposal seem to be inspired in the same strategy since we also project the points to extract the approximate 2D wall’s location. Nevertheless, this is only used to later retrieve the corresponding 3D data and delimitate with precision the points belonging to the walls.

This work aims to make progress in the creation of automatic 3D models from point clouds provided by scanners [21]. The contributions of this work lie in three points: accuracy of the model, coherent representation and evaluation of the method. First, we propose a voxelization (discretization) of the space with which to accurately define the planes fitted to the points belonging to the ceiling, floor and walls of the facility. Second, we generate a complete boundary representation model of the indoor in which faces, edges and vertices are coherently connected. Third, once the method is designed and implemented, we make a performance evaluation by measuring the geometric modeling accuracy, the recognition accuracy and the relationship modeling accuracy. The following sections explain the main stages to achieve a complete B-rep model of interiors and present the results obtained.

## 2. 3D Data Segmentation

### 2.1. Floor and Ceiling Segmentation

The first stage in the 3D model creation process consists of efficiently segmenting the data obtained by the scanner from different positions. Here we assume that preprocessing stages like outliers/noise filtering and data registration are done, so that our input is an unstructured large cloud of points.

One of the problems which arise in the automatic walls detection is the voxelization of the space in the sense of which the best voxels’ size is and where the origin of the space is set. In [18,19] this aspect is not tackled. In the present work, we deal with the optimization of the voxels’ size, allowing us to adjust more precisely and at the same time, the ceiling, floor and walls of rectangular rooms into the voxel planes. Thus, a voxel plane contains the majority of the data of each wall.

First of all, we face up the identification and segmentation of the floor and the ceiling of the room. The method proposed in this section assumes that, as it is usual in construction, floor and ceiling are parallel structures. From here on the word “wall” will be indistinctly used to refer any flat indoor structure (ceiling, floor or wall). Our approach is based on creating an optimum discretization of the space (from here on called “voxel space”) and then accurately defining the planes which contain the maximum number of points which lie in the floor and ceiling.

Formally, the voxel space can be defined in a universal coordinate system (UCS) by means of the voxel size (*ε*, *δ*, *σ*) and the coordinates of centroid’s voxel (*v_x_*, *v_y_*, *v_z_*), *v_z_* being voxel’s height, according to the construction context.

**Figure 1.**
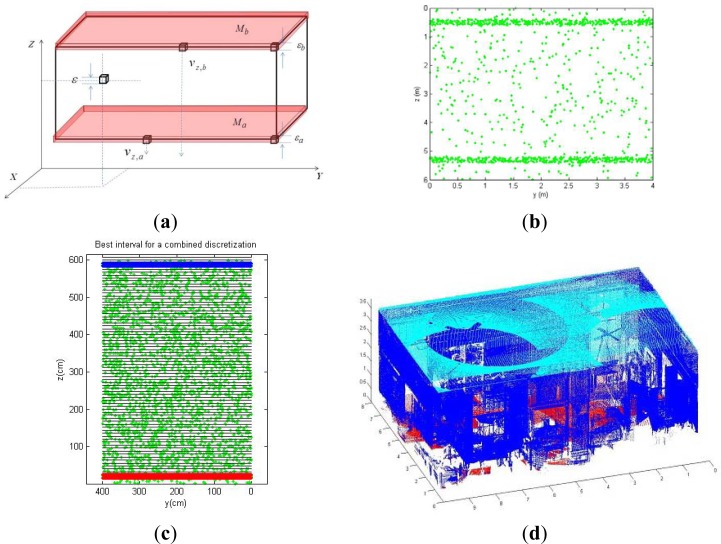
(**a**) Representation of the voxelized space. (**b**) and (**c**) Simulated data of the interior of a room and final discretization of the space. Blue and red planes of voxels containing the majority of the points of floor and ceiling. (**d**) Real segmentation. Point cloud sensed in a room and segmented points belonging to ceiling (cyan) and floor (blue).

Assuming cubic voxels, our objective is then addressed on determining the minimum voxel size, characterized by the parameter *ε*, and the first coordinate of the voxel plane *v_z_* which contains the maximum number of the points belonging to a wall. Once the voxelization of the space is carried out, most of the points belonging, for example, to the floor are contained in a narrow parallelepiped *M* whose height is *ε* (see Figure 1(a)). The uncertainty in *ε* can be limited by means of the flatness specifications provided in the construction standards.

The trade-off between the size of the voxels and the number of points contained in *M* is regulated by the objective function (1), in which *p(ε*, *v_z_)* is the percentage of the data contained in *M.* Therefore, this function evaluates the percentage of the wall’s data contained in a voxel plane *versus* the voxel’s size. Figure 1 shows the voxelization of the space according to the definition of the volume *M* for the floor (*M_a_*) and the ceiling (*M_b_*):

(1) $$F(\varepsilon ,v_{z})=\frac{p^{2}(\varepsilon ,v_{z})}{\varepsilon }$$

In order to obtain the maximum value of function *F*, attaining the maximum number of the wall’s data inside a voxel plane, different restricted ranges are defined for variables *ε* and *v_z_*. On the one hand, *ε* is delimited by the range [*ε_1_*, *ε_2_*] in which *ε_1_* corresponds to the precision of the scanner (in our case, 1 cm) and*ε_2_* is determined by the flatness tolerance, allowed in the international specification DIN 18202.

On the other hand, *v_z_* is initialized in the position *m*, which corresponds to the maximum value of the data distribution around the wall. For each value of *ε* in the range [*ε_1_*, *ε_2_*] the value of *v_z_* is evaluated for the range [*m* − *ε*, *m + ε*] and function *F* is then calculated. Once this process is finished, the maximum value of *F* (*max F* in Algorithm 1) provides the optimum values *ε’* and *v’_z_* respectively. The algorithm is shown below.

When the algorithm is separately applied for ceiling and floor, two different voxel space configurations are generated. Let (*v’_z,a_*, *ε’_a_*) and (*v’_z,b_*, *ε’_b_*) be the position and voxel size parameters calculated for the floor and the ceiling of the room. We propose a new function *G* which integrates both voxelization proposals.

**Algorithm 1.** Calculation of optimum values *ε’* and *v’_z_*.

*F*(*ε*,*v_z_*)← 3D data distribution in axis Z
*m←* fitted Gaussian function
*max F← F(ε_1_,m)*
**for each** *ε***←***ε*_1_ to *ε*_2_**do**
**for each** *z***←***m-ε* to *m+ε* **do**
*F*(*ε*,*z*)
**if***F*(*ε*,*z*) > *max F* **then**
*max F← F*(*ε*,*z*); *ε’ ← ε ; v’_z_← z*
**end**
**end**
**end**

Equation (2) imposes one unique voxel size *E* and the positions of the planes of voxels *V’_z,a_* and *V’_z,b_*, attaining the maximum value of G which provide us the best simultaneous planes of voxels for ceiling and floor. The new proposed function to optimize is as follows:

(2) $$\begin{array}{c} {\text{argmax}}_{\Omega }\{G\left(E,V_{z,a},V_{z,b}\right)\}\\ \Omega =E\in [{\varepsilon^{\prime }}_{1},{\varepsilon^{\prime }}_{1}],V_{z,a}\in [v_{z,a}-\frac{{\varepsilon^{\prime }}_{a}}{2},v_{z,a}+\frac{{\varepsilon^{\prime }}_{a}}{2}],V_{z,b}\in [v_{z,b}^{,}-\frac{{\varepsilon^{\prime }}_{a}}{2},v_{z,b}^{,}+\frac{{\varepsilon^{\prime }}_{a}}{2}],\\ G\left\{E,V_{z,a},V_{z,b}\right\}=p_{a}\left(E,V_{z,a}\right)+p_{b}\left(E,V_{z,b}\right) \end{array}$$

*p_a_* and *p_b_* are the occupation percentages for the parameters *ε’_a_* and *ε’_b_*. The pseudocode of the algorithm which obtains *G* is detailed in Algorithm 2.

**Algorithm 2.** Obtaining the function *G*.

**for each** *ε ← ε*_1_ to *ε*_2_ **do**
**for each** *z ← z_a_−ε/2* to *z_a_+ε/2* **do**
calculate *ceiling voxel centroid b*
calculate number of points in intervals *a* and *b*
calculate percentage of points *p_a_* and *p_b_*
**end**
**for each** *z ← z_b_−ε/2* to *z_b_+ε/2* **do**
calculate *ceiling voxel centroid a*
calculate number of points in intervals *a* and *b*
calculate percentage of points *p_a_* and *p_b_*
**end**
**end**
*G = maximum(sum(p_a_,p_b_))*

### 2.2. Performance Tests

The approach detailed above has been tested in simulated and real data. Figure 1 shows an example of the results obtained under simulation. In Figure 1(b) a front view of a simulated point cloud of a room is depicted. It can be seen two dense point regions, which correspond to the floor and ceiling, and sparse regions which simulate the rest of the sensed points in the room. The maximum values of two Gaussian functions fitted to the data distribution determine the initial values of *v_z,a_* and *v_z,b_*. Figure 1(c) shows the voxels planes projected over the plane YZ, the 3D data and two slices in red and blue that contain the majority of the sensed points. The red voxels plane contains points of the floor and the blue one contains points of the ceiling. We tested the algorithm over twenty simulated rooms. The occupation percentage averages for ceiling and floor were 96.9% and 92.1% respectively.

Figure 1(d) presents the result for a real case. We illustrate the 3D data sensed by a laser scanner from five positions of an inhabited classroom. The data segments corresponding to the ceiling and floor are painted in cyan and red. The total point cloud was composed by 1.5 million points, and the size of the segmented regions was 187,000 points for the ceiling and 95,000 points for the floor.

### 2.3. Walls Segmentation

#### 2.3.1. Rectangular Indoor Plans

In this section we present an approach to segment 3D data corresponding to each one of the walls of a rectangular indoor plan. A rectangular plan is the easiest case to be dealt with. The strategy explained in Section 2.1 can here be extended by considering three pairs of parallel voxels planes, so that the Equation (2) can be easily extended. The objective is to find six parallelepipeds (slices) with centers *c_i_*, *i = 1,…6* and with a common width *ε* which contain the maximum number of points belonging to the walls of the room. Formally, the objective is:

(3) $$\begin{array}{c} \underset{\varepsilon ,c_{i}}{\text{argmax}(G)}\\ G(\varepsilon ,c_{1},c_{2},...,c_{6})=\sum_{i=1}^{6}p_{i}(\varepsilon ,c_{i})\;\;\;\;\varepsilon \in \left[{\varepsilon^{\prime }}_{\text{min}},{\varepsilon^{\prime }}_{\text{max}}\right],c_{i}\in \left[z_{i}-\frac{{\varepsilon^{\prime }}_{i}}{2},z_{i}+\frac{{\varepsilon^{\prime }}_{i}}{2}\right],i=1...6 \end{array}$$

#### 2.3.2. Arbitrary Indoor Plans

**Figure 2.**
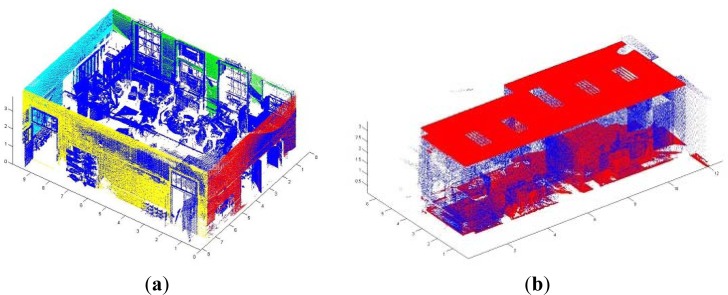
Segmentation of floor and ceiling in rectangular (**a**) and arbitrary (**b**) plans. (a) shows the segments of the four walls of the rectangular indoor presented in Figure 1(d).

Assuming that floor and ceiling lie in parallel planes, the approach proposed in Section 2.1 can always be used to detect the floor and the ceiling in any non-rectangular room of a building. Figure 2(b) illustrates the extraction of the points belonging to the floor and ceiling of an arbitrary plan. However, the identification of the walls is a more complex task.

**Figure 3.**
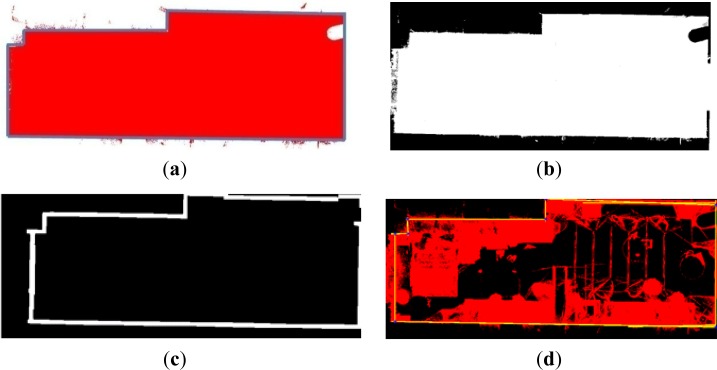
Stages in a wall segmentation process. (**a**) Visualization of the point cloud from a zenital viewpoint. (**b**) Binary image generated after discretization. (**c**) Boundary detection. (**d**) Defining edges and corners in the image.

The projection of the 3D data from a specific viewpoint allows us to obtain a normalized binary image in which each pixel can be occupied by one or more 3D points (white pixels in Figure 3(b)) or not. From here on, we will denote *I* the projected image of the data from a top view. This image will help us to obtain a coarse location and position of the walls which will be later refined. The segmentation process is as follows.

After creating the image *I*, the boundary of the room is extracted and, through a Hough Transform algorithm, the set of edges corresponding to the walls in the projected image are detected in a 2D context. As the reader may suppose, if a wall or the connectivity between two walls is completely occluded by a piece of furniture or a constructive component, the boundary of the room does not fit to the walls but these components. Once the room boundaries are demarcated, the points out of the boundaries are removed automatically. Afterwards, we figure out the intersections between edges and obtain the corners in the image. Figure 3 shows the steps of the segmentation process: (a) 3D point cloud viewed from the top of the room; (b) Discretization of the view and generation of binary image *I*; (c) Boundary extraction in *I.* (d) Edge and corner detection.

**Figure 4.**
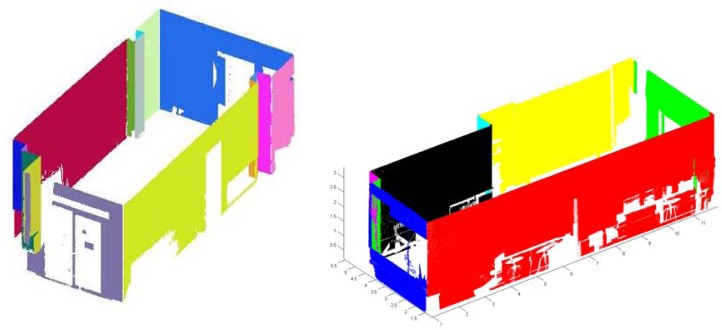
Retrieval of 3D points corresponding to the walls.

Assuming vertical walls, the segments and corners in the image signify planes and edges in the 3D context. And the planes will be used to segment the 3D points which lie into each wall. Thus, we calculate the mean square distance of the point cloud to the walls and classify each point into a wall. Figure 4 shows the segmentation of points belonging to the walls for two different rooms.

## 3. Creation of B-Rep Models

The segmentation stage provides the set of points belonging to each wall (including floor and ceiling) of the indoor scene. The following step consists of converting this raw information into high level surface representation. Within the 3D representation models universe we have chosen the boundary representation (B-rep) model [22]. In B-rep, a shape is described by a set of surface elements along with the connectivity information which describe the topological relationship between the elements.

The process to achieve a B-rep of an interior space starts calculating the planes which best fit every set of the segments associated to the walls. To do this, we have used the Singular Value Decomposition (SVD) technique [23]. Through SVD, the closeness between the plane and the segmented points is easily calculated. The steps to calculate the plane equation that best fits a generic set of points are shown below.

Each point cloud *P = (p_1_,p_2_,…,p_n_)*, corresponding to a wall, can be fit to a plane defined by the equation:

(4) Π:A x+B y+C z+D=0

The best fit plane is that which minimizes the sum of the distances between every point *p_i_* and the plane *Π*. Therefore, we can calculate each fit plane by minimizing the expression:

(5) $$\sum_{i=1}^{n}\frac{{\left|Ax_{i}+By_{i}+Cz_{i}+D\right|}^{2}}{A^{2}+B^{2}+C^{2}}$$

Setting the partial derivative with respect to *D* equal to zero, we obtain:

(6) $$D=-\left(Ax_{0}+By_{0}+Cz_{0}\right)$$

in which *p_0_= (x_0_, y_0,_z_0_)* is the centroid of *P*. Replacing (6) in (5):

(7) $$\sum_{i=1}^{n}\frac{{\left|A\left(x_{i}-x_{0}\right)+B\left(y_{i}-y_{0}\right)+C\left(z_{i}-z_{0}\right)\right|}^{2}}{A^{2}+B^{2}+C^{2}}$$

Let us introduce the matrix *M* = [*p_1_ − p_0_ p_2_ − p_0 …_ p_n_ − p_0_*]*^T^*, in which *p_i_= (x_i_, y_i_, z_i_)* and *p_0_= (x_0_, y_0_, z_0_)*, and the vector *v =* [*A  B  C*]*^T^*. We can show the problem over a matrix representation:

(8) $$\frac{\left(v^{T}M^{T}\right)\left(Mv\right)}{v^{T}v}$$

This expression is called a Rayleigh Quotient and is minimized by the eigenvector of *M^T^M* that corresponds to its smallest eigenvalue. Next, we use the singular decomposition of *M* = *USV^T^*, in which the columns of *V* are the singular vectors of *M* and the eigenvectors of *M^T^M*. Therefore, the solution of Equation (5), provide us the normal of the plane *Π*, $\vec{n_{\Pi }}=[\begin{array}{ccc} A & B & C \end{array}]$. The parameter *D* is calculated from Equation (6).

The last stage consists of calculating the intersections between connected planes. Note that the topological relationship between the walls is established as the edges and corners are extracted in figure *I*. So, we know which planes have to be themselves intersected and, therefore, we can find the 3D edges and corners of the room. For instance, the ceiling and floor’s planes pairwise intersect with each wall’s plane and define the edges at the top and down of the room; and vertices are extracted after intersecting three planes.

**Figure 5.**
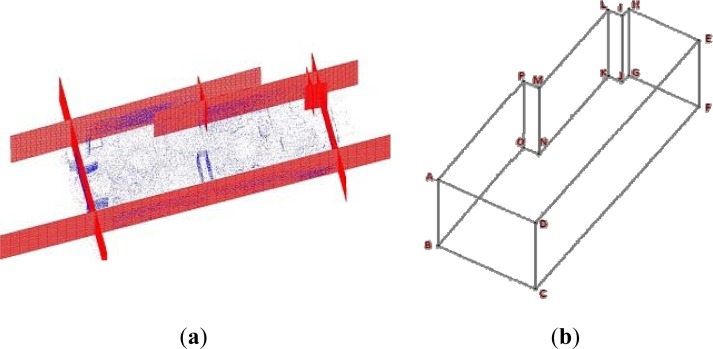

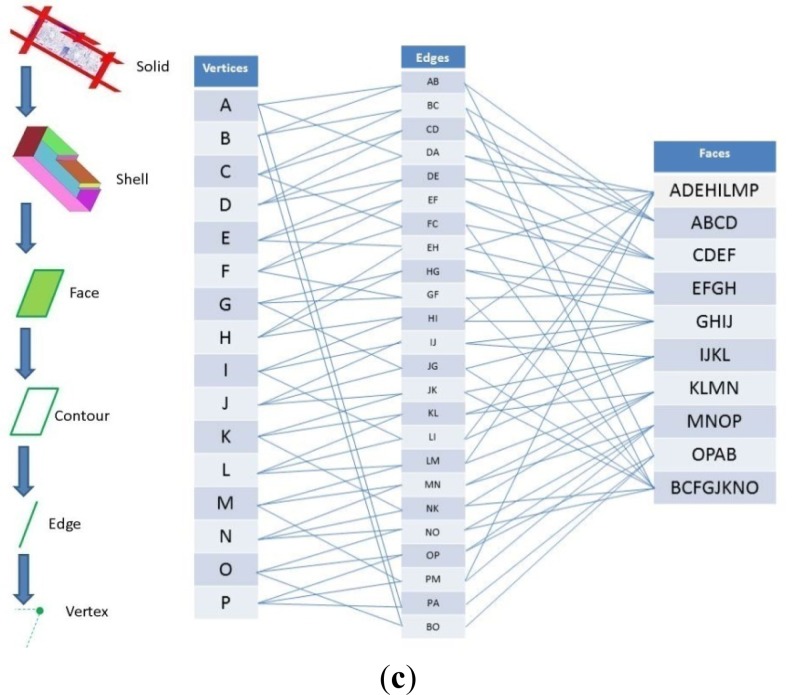
(**a**) Planes fitting the walls. Note that the planes (in red) do not represent the walls but the planes which fit the walls. They are merely used to illustrate the intersections of such planes. (**b**) Labeled vertices of the room. (**c**) Decomposition of a solid in simple objects. Relationship between topological elements in the test room.

Figure 5 illustrates an example with the results of our approach. Part (a) represents the planes fitted to the walls and part (b) shows the set of labeled vertices of the room. Figure 4(c) contains the relationship graph in which adjacent faces in the diagram share one edge.

## 4. Results

Our approach was tested on panoramic range data appertaining to inhabited interiors. Note that we are dealing with very complex scenarios, in which furniture and other objects contribute to clutter and occlusions. They contain a wide variety of objects that occlude not only the walls, but also the ceiling and floor. Three to six laser scans were taken per room. A FARO Photon laser scanner provided 38 million points per room. Figures 7 and 8 show the resulting 3D B-rep models for two rooms.

In this section we present the results obtained for two different inhabited indoors. These interiors do not have rectangular but arbitrary plans. The first room corresponds to the Virtual Reality Lab at the Escuela Técnica Superior de Ingenieros Industriales (UCLM). The second one is the living room of a private flat.

**Figure 6.**
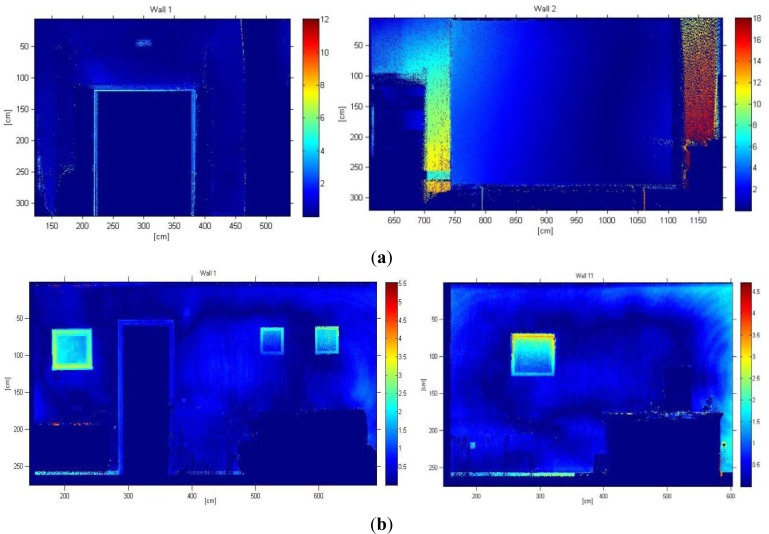
Deviation of the data to the fitted planes for two walls of the lab (**a**) and two walls of the living room. (**b**) Colorbars are coded in centimeters.

After defining the B-rep model we aim to investigate the accuracy of the obtained models. Firstly, we determine the error committed when we represent the walls of the rooms by means of planes. We thus measure the quadratic distance of every point of the walls to the corresponding plane. Distances between the sensed points and the planes are represented in colormaps in Figure 6(a) for different walls of the lab and (b) for two walls belonging to the living room. It can be seen different regions where an important variation of color is produced. These areas can be owing to typical objects hanging on the wall (pictures, posters, and so on), moldings and doorframes. Of course, some of the errors come from the fact that the walls, ceiling and floor are not totally flat. The corresponding error means, for both inhabited interiors, are presented in Table 1, in which *d̄* represents the mean value for the distances between each 3D point and its corresponding plane.

The mean error of the fitted planes ranged between 0.59 and 4.64 cm for the lab and between 0.19 and 6.5 cm for the room. In any case, for the majority of the walls the mean error is around below 2 centimeters, despite being severely occluded by tables and chairs. On the other hand, the wall which fitted worse was number 2 of the lab. In this case, there was a big projection panel which largely occluded the wall. Most points sensed in this part of the room corresponded to the panel, so that the calculated plane fitted the panel instead of fitting the wall.

We focused most of our analysis on understanding the performance of our modeling results, since this aspect of the algorithm is considerably less studied than planar wall modeling. We considered two aspects of the performance: first, how reliably the walls can be detected, and second, how accurately they are modeled. To answer the first question, we compared the detected walls with walls of the ground truth model. No fails were reported in this aspect and all existing walls were correctly detected. Failed detections mainly might occur in severe occlusion circumstances.

**Figure 7.**
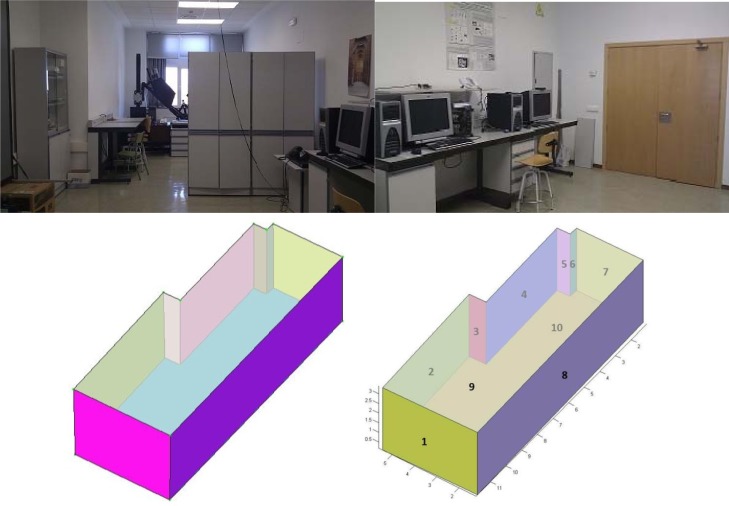
Images of the tested lab (**top**). Our model and the ground truth model (**bottom**).

**Figure 8.**
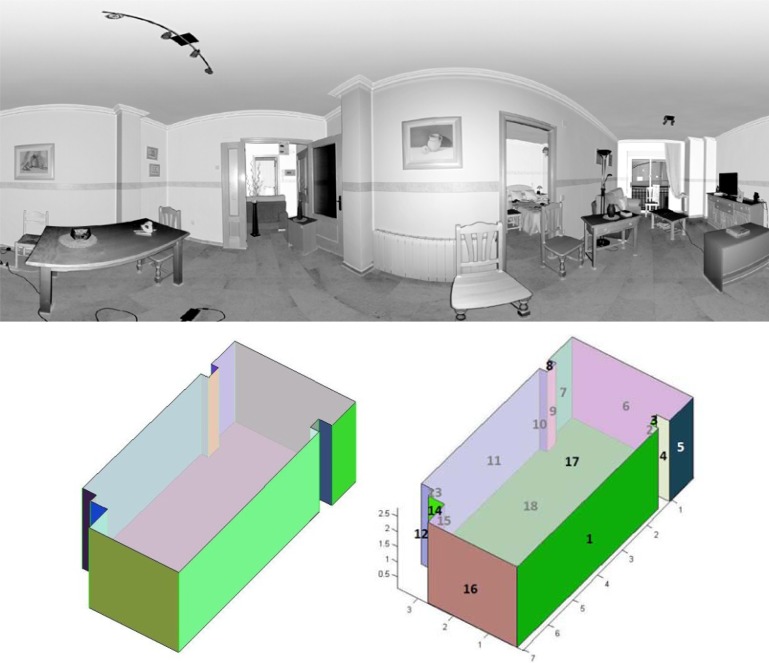
Planar image of the living room (**top**). Our model and the ground truth model (**bottom**).

The second question was tackled first generating the geometrical ground truth of the scenes. We constructed by hand the ground truth models of the rooms with the help of a Leica DISTOTM A6 laser tape measure, which provides 1 millimeter accuracy. In order to assess the error committed, the ground truth models were then compared with our 3D models (see Figures 7 and 8). The results are summarized in Table 2.

We first compared the lengths of the vertical and horizontal edges of each face of the ground truth models with ours and the difference between are denoted as *d_v_* and *d_h_* in Table 2. The value of *d_v_* was similar for all pairs of walls; around 0.87 cm for the lab and 0.80 cm for the living room. The smallest value of *d_h_* in the living room was for number 3. In this case, the mean value was less than 2 cm in both rooms.

In order to compare the accuracy of the orientation of the faces, we calculated the difference between the respective normal vectors (*α* in Table 2). The smallest faces yielded a high rate in *α*, which distorted the average value. Thus, although the mean value of *α* were 1.59° and 1.85° for the lab and the living room respectively, in the majority of the faces *α* was less than this value.

Once we have presented the results for these two inhabited interiors, we can establish some difference between them. The living room has more and smaller walls than the laboratory, what might lead the reader to believe that the segmentation process is particularly complicated in this case. However, if we compare the mean values for the different parameters in Tables 1 and 2, the deviations with the ground truth are lower in the living room’s case. This result can be due to the fact that a multitude of pieces of furniture occlude the walls of the room and, consequently, the segmentation process may yield more imprecise segments.

## 5. Conclusions

Automatic creation of “as-is models” of inhabited scenarios is a challenge research field which is gaining attention from different applications in AEC/FM contexts. In this paper an approach for automatically creating Boundary Representation Models (B-rep) from dense point clouds collected by laser scanners is presented.

The method here proposed is based on segmenting the data by optimizing the discretization of the space. We make a 2D projection of the data in the voxel space and coarsely determine the segments in which the points lie. The voxels’ size is adjusted to the walls, taking into account the flatness tolerances in construction, improving the approach of previous works [18,19] in which predefined voxels’ sizes were used. Then the boundary representation model is generated by intersecting the planes that contains the 3D segments, calculating the faces, edges and vertices, and establishing the relationship between components. This idea has been tested in arbitrary shape plans providing excellent results.

The results lead us to state that the majority of the wall’s points were correctly segmented. We have also evaluated the performance and accuracy of our method comparing the ground truth and the query B-rep models. Overall modeling accuracy for the AEC domain typically needs to be at least within 2.5 cm of the ground truth, so that the precision of our model is clearly above the standard (between 0.8 cm and 1.88 cm for vertical and horizontal edges).

This research is a part of a larger project which aims to obtain automated reverse engineering of buildings including more complex semantic models. Future improvements to the method will be addressed in two lines: extend the method to non-flat walls and generate B-rep models including details (paintings, panels, *etc.*) and parts of the walls (moldings, doorframes, windows frames, *etc.*) in inhabited buildings. As other potential applications, these 3D models can be helpful to create, in an automatic manner, virtual scenes in which digitized objects are introduced in order to generate virtual exhibitions as the ones shown in [24–26].

## Figures and Tables

**Table 1.** Mean deviation of the scanned data to fitted walls.

**Lab**	**Wall 1**	**Wall 2**	**Wall 3**	**Wall 4**	**Wall 5**	**Wall 6**	**Wall 7**	**Wall 8**	**Mean**
*d̄* [cm]	1.13	4.64	1.71	0.59	0.81	1.44	1.23	1.16	**1.58**

**Living room**	**Wall 1**	**Wall 2**	**Wall 3**	**Wall 4**	**Wall 5**	**Wall 6**	**Wall 7**	**Wall 8**	**Wall 9**	**Wall 10**	**Wall 11**	**Wall 12**	**Wall 13**	**Wall 14**	**Wall 15**	**Wall 16**	**Mean**
*d̄* [cm]	0.55	1.11	0.82	0.19	6.52	4.60	0.24	1.86	0.33	0.57	0.49	4.11	0.44	0.4	0.28	1.08	**1.47**

**Table 2.** Parameters calculated for each pair of walls.

**Lab**	**Wall 1**	**Wall 2**	**Wall 3**	**Wall 4**	**Wall 5**	**Wall 6**	**Wall 7**	**Wall 8**	**Wall 9**	**Wall 10**	**Mean**
α (º)	1.09	0.09	1.12	1.58	6.62	4.83	0.08	0.45	-	-	**1.59**
*d_v_*(*cm*)	0.81	0.80	0.89	0.90	0.90	0.89	0.89	0.90	-	-	**0.87**
*d_h_*(*cm*)	0.52	3.21	1.83	1.96	1.12	0.60	0.98	4.86	-	-	**1.88**

**Living room**	**Wall 1**	**Wall 2**	**Wall 3**	**Wall 4**	**Wall 5**	**Wall 6**	**Wall 7**	**Wall 8**	**Wall 9**	**Wall 10**	**Wall 11**	**Wall 12**	**Wall 13**	**Wall 14**	**Wall 15**	**Wall 16**	**Wall 17**	**Wall 18**	**Mean**
α (º)	2.25	10.3	0.95	2.49	0.12	0.72	6.62	0.16	3.86	2.55	1.12	0.89	0.58	0.20	0.11	0.56	-	-	**1.85**
*d_v_*(*cm*)	0.80	0.79	0.79	0.80	0.79	0.79	0.80	0.80	0.80	0.80	0.80	0.80	0.80	0.80	0.80	0.80	-	-	**0.80**
*d_h_*(*_cm_*)	0.74	0.39	0.09	0.99	0.15	1.80	0.11	2.05	0.32	1.46	2.18	1.31	1.29	2.15	1.17	4.45	-	-	**1.29**
